# Variations in Intestinal Microbiota Among Three Species in the Cervidae Family Under the Same Feeding Conditions

**DOI:** 10.3390/vetsci12050438

**Published:** 2025-05-03

**Authors:** Yichen Wang, Minghui Shi, Jiahui Wu, Xiangyu Han, Mengqi Li, Yining Wu, Yuanlin Jiang, Haonan Zhang, Shuqiang Liu, Defu Hu

**Affiliations:** School of Ecology and Nature Conservation, Beijing Forestry University, Beijing 100083, Chinaliushuqiang@bjfu.edu.cn (S.L.)

**Keywords:** sika deer (*Cervus nippon*), red deer (*Cervus elaphus*), white-lipped deer (*Cervus albirostris*), gut microecology, high-throughput sequencing

## Abstract

The breeding of large animals in the family Cervidae in China contributes to achieving two tasks: restoring the provenance of wild populations and providing raw materials for traditional Chinese medicine. Currently, red deer (*Cervus elaphus*), sika deer (*C. nippon*), and white-lipped deer (*C. albirostris*) maintain a large number of breeding populations. Some studies on the relationship between the intestinal microbiota and the feed of these deer have been conducted; however, owing to differences in feeding conditions between studies, it has been impossible to compare the intestinal microecology and related adaptability between species. Therefore, the present study is aiming to investigate whether the differences in intestinal microbiota of the three deer species are related to the distance of phylogenetic relationships under the same feeding environment. On this basis, we discuss whether there are differences in the adaptability of the intestinal microbiota of the three deer species to feed nutrients, deepen the understanding of the relationship between the three deer intestinal microbiota and feed nutrition, and provide basic data for improving the scientific feeding of the three deer species. In this study, 16S rRNA high-throughput sequencing technology was utilized to analyze the intestinal microbiota in feces of the abovementioned healthy deer species. The results of this study indicated that the intestinal microbiota diversity and relative abundance in female white-lipped deer (FWLD) were significantly lower than those in female sika deer (FSD) and female red deer (FRD; *p* < 0.05); however, there was no significant difference between the latter two groups (*p* > 0.05). The community compositions of the intestinal microbiota in FSD and FRD were more similar, whereas that of FWLD was significantly different from those of the first two groups. Firmicutes and Bacteroidetes were the most abundant phyla in the intestinal microbiota of all three deer species, and *Ruminococcceae_UCG-005* was the most abundant genus. No known obligatory pathogenic bacteria were observed in any sample. The relative abundance of the operational taxonomic units *Christensenellaceae_R-7_group*, *Treponema_2*, and *Akkermansia* exhibited significant differences among FSD, FRD, and FWLD, respectively. Therefore, the phylogenetic relatedness of the three deer species appears to play a major role in their intestinal microecology under the same feeding conditions—the greater the phylogenetic relatedness between hosts, the more similar is their intestinal microbiota. In addition, the PICRUSt (Phylogenetic Investigation of Communities by Reconstruction of Unobserved States) function prediction results indicated that FSD were less capable than FRD and FWLD in the functional category of nutrient metabolism, and FWLD were less capable than FSD and FRD in the functional category of intestinal absorption. These results indicated that there may be differences in the nutritional adaptation abilities of the three deer species under different feeding conditions. In summary, these results revealed the differences in intestinal microbiota among the three deer species under the same food conditions, indicating that the intestinal microbiota of the three deer species had significant differences in food adaptation. Based on this, the nutritional supply of feed for the three deer should consider the species differences.

## Simple Summary

The intestinal microbiota structure and diversity of sika deer, red deer, and white-lipped deer in Liaoning were analyzed using 16S rRNA high-throughput sequencing technology. The study indicated that the intestinal microbiota diversity and relative abundance in female white-lipped deer were significantly lower than those in female sika deer and female red deer; however, there was no significant difference between the latter two groups. In summary, these findings can provide a scientific basis for the feeding and management of three deer species to improve their health.

## 1. Introduction

The intestinal microbiota of an animal refers to all bacterial communities in the small and large intestines of the host, including symbiotic and commensal bacteria, conditional pathogens, and pathogenic bacteria. They exhibit mutualistic symbioses and interact with the host to develop an intestinal microecosystem [1]. The intestinal microecosystem is a dynamically balanced system closely related to many physiological processes in the host, such as digestive physiology [2], metabolism [3], immune system regulation [4], and resistance to pathogens [5]. Owing to structural differences in digestive system, ruminants and monogastric animals exhibit distinct characteristics of intestinal microbiota. Ruminants rely on microbial fermentation, whereas monogastric animals primarily utilize enzymatic digestion, and monogastric animals have simpler stomach structures, except for horses and rabbits, where both processes coexist [6]. Moreover, digesta retention time differs substantially between ruminant and monogastric digestive systems, directly influencing microbial fermentation efficiency. Ruminants demonstrate prolonged retention (30–50 h) in the rumen prior to intestinal passage, enabling thorough fiber degradation through sustained microbial activity [7]. Conversely, monogastric animals exhibit significantly shorter gastrointestinal transit times, consequently restricting microbial-mediated digestion. Distinct from rumen and intestinal contents, feces are typically used to represent the overall metabolic function and microbial composition of an animal’s intestine in many studies [8]. For example, variations in fecal microbiota can affect nutrient absorption and environmental adaptation [9]. Additionally, it has been demonstrated that the composition of fecal microbiota reflects the composition and function of the intestinal bacteria [10]. These findings suggest that using feces samples to understand the composition and diversity of ruminant microbial communities is both effective and convenient.

Numerous studies have found that the composition and dynamics of the intestinal microbiota among individuals of the same species are affected by multiple factors, such as their living environment [11], diet [12], age [13], and sex [14]. Diet composition plays an especially important role in the species composition and dynamics of the intestinal microbiota. Even different species of the same trophic level consuming the same foods have very similar compositions of intestinal microbiota [15,16,17]. All intestinal microbiota have species-specific characteristics, that is, the genetic characteristics of their hosts play an important role in the composition of the intestinal microbiota. However, few comparative studies have been conducted on the intestinal microbiota of closely related species under identical conditions [18,19]. For ex situ, protected, closely related species that are of the same trophic level, the relationship between the host genetic characteristics and intestinal microbiota is the scientific basis for formulating an appropriate feed supply and maintaining good nutritional physiological processes.

Sika deer (*Cervus nippon*) are primarily distributed in the eastern China monsoon area, Russian Far East, and Japan. Red deer (*C. elaphus*) are primarily distributed in the mountainous forests of northern Eurasia. White-lipped deer (*C. albirostris*) are primarily distributed in the forest areas of the eastern Qinghai–Tibet Plateau in China. All three species are large deer and exhibit browsing behavior. They are all resource animals with high edible and medicinal value and are bred to maintain populations in many parts of China. These three species have been artificially bred for reintroduction in accordance with the Endangered Wildlife Conservation Plan formulated by the National Forestry and Grassland Administration. The breeding of these species is not only industrially valuable but is also significant for conservation biology. Currently, in the wild, China has fewer than 1500 sika deer [20], approximately 150,000 red deer [21], and about 15,800 white-lipped deer [22]. Under captive conditions, there are about 1.5 million sika deer [23], roughly 100,000 red deer [24], and only fewer than 500 white-lipped deer for conservation and research purposes. Understanding the correlation between the host genetic characteristics and intestinal microbiota of these species provides a foundation for the development of feeding, breeding, and management strategies. Several studies have been conducted on the intestinal microbiota of *C. nippon* [25,26], *C. elaphus* [27,28], and *C. albirostris* [29,30,31,32]. However, owing to their different geographical and dietary conditions, it was impossible to analyze the relationship between the genetic characteristics and intestinal microbiota of the three deer species.

In this study, healthy adult sika deer, red deer, and white-lipped deer were chosen as experimental animals which were raised under the same living conditions. We utilized 16S rRNA high-throughput sequencing technology to explore the intestinal microecosystems of the three deer species. This study aims to investigate whether the differences in intestinal microbiota of the three deer species are related to the phylogenetic distance under the same feeding environment. On this basis, we discuss whether there are differences in the adaptability of the intestinal microbiota of the three deer species to feed nutrients, deepen the understanding of the relationship between the three deer intestinal microbiota and feed nutrition, and provide basic data for improving the scientific feeding of the three deer species.

## 2. Materials and Methods

### 2.1. Sample Collection and Preservation

Samples were collected from the National Deer Provenance Base of Liaoyang Qianshan Chenglong Technology Co., Ltd. in Liaoyang, Liaoning Province, China, in April 2021. Sika deer, red deer, and white-lipped deer were raised together on the same farm. Sika deer and red deer were kept separately by species and sex, with about 30–50 individuals per group. White-lipped deer were housed in mixed-sex groups. Thirty healthy adult deer of similar ages were selected: 10 female sika deer (FSD), 10 female red deer (FRD), and 10 female white-lipped deer (FWLD). None of the individuals had been administered antibiotics in the past three months. Fecal samples were collected from each individual between 06:00 and 08:00 BJT on consecutive days. Sampling lasted for 3 days, and samples were taken once a day for a total of 3 times. Then, three samples were mixed to produce a single sample for each individual. During observations of the experimental populations, fresh feces were collected in sterile self-sealing bags immediately following defecation, labeled by individual, and stored at −20 °C. During the sampling period, all deer were fed corn straw silage and a small amount of concentrated feed (Table 1), with sufficient drinking water. No signs of disease were observed in any of the experimental individuals during the sampling period.

### 2.2. DNA Extraction, PCR Amplification, and Sequencing

Total DNA was extracted from the fecal samples using an MN NucleoSpin^®^ 96 Soil Kit (Macherey-Nagel GmbH & Co. KG, Düren, Germany) as per manufacturer instructions. The concentration, total quantity, purity, and band integrity of the DNA samples were determined via 1.0% agarose gel electrophoresis. Samples of sufficient quality and quantity were preserved at −80 °C.

The V3-V4 region of the bacterial 16S rRNA gene was amplified via polymerase chain reaction (PCR) utilizing the primers 338F (5′-ACTCCTACGGGAGGCAGCA-3′) and 806R (5’-GGACTACHVGGGTWTCTAAT-3’). The reactants utilized for PCR amplification were as follows: 200 ng DNA samples, 1.5 μL forward primer (10 μmol/L), 1.5 μL reverse primer (10 μmol/L), 25 μL 2 × PCR Buffer for KOD FX Neo (TOYOBO BIOTECH Co., Ltd., Shanghai, China), 10 μL dNTP (2 mmol/L), 1 μL KOD FX Neo (1 U/μL; TOYOBO BIOTECH Co., Ltd., Shanghai, China), and ddH2O to supplement the total reaction volume to 50 μL. The reaction conditions were as follows: 95 °C for 5 min (initial denaturing), followed by 25 cycles of 95 °C for 30 s (denaturing), 50 °C for 30 s (annealing), 72 °C for 40 s (extension), and 72 °C for 7 min (final extension). The amplicons were preserved at 4 °C.

Amplicons were quantified using 1.8% agarose gel electrophoresis and mixed in a 1:1 mass ratio. Mixed amplicons were then purified using an E.Z.N.A.^®^ Gel & PCR Clean Up Kit (OMEGA, Norcross, GA, USA). Eventually, the final amplicons were excised and recovered using a Monarch^®^ DNA Gel Extraction Kit (NEB, Ipswich, MA, USA). The constructed library was subjected to quality inspection using Qsep-400. After library construction, the recovered amplicons were sequenced using an Illumina HiSeq 2500 platform (Illumina, Inc., San Diego, CA, USA). Library construction and sequencing work were entrusted to Biomaker Technologies Co., Ltd. in Beijing, China. The sequencing data generated in our study have been deposited in the SRA database.

### 2.3. Bioinformatics Analysis

Raw sequencing data were filtered using Trimmomatic version 0.33 [33]. The identification and removal of primer sequences was processed using Cutadapt version 1.9.1 [34]. Paired-end reads were concatenated using USEARCH version 10 [35]. Finally, chimeric sequences were removed using UCHIME version 4.2 [36]. The remaining effective reads were clustered into operational taxonomic units (OTUs) at a 97% sequence similarity threshold using USEARCH [35,37]. Utilizing SILVA (Release 132, http://www.arbsilva.de, accessed on 10 November 2023) as a reference database, feature sequences were annotated using a naïve Bayes classifier to determine the community composition of samples at each taxonomic rank. Species abundance tables for different taxonomic ranks were constructed using QIIME2. Alpha diversity was analyzed using QIIME2, and beta diversity was analyzed using QIIME to compare microbiota abundance and diversity among the different groups. Community composition figures, Non-metric Multidimensional Scaling (NMDS) analyses, principal coordinate analyses (PCoA), and Analysis of Similarities (ANOSIM) were performed using the ecodist and vegan packages in R (version 4.1.3; R Core Team 2023). All figures, including community composition figures, were created using the ggplot2 package in R. Linear Discriminant Analysis (LDA) Effect Sizes (LEfSe) were calculated to determine the statistical differences in microbiota species composition among the three host species. Phylogenetic Investigation of Communities by Reconstruction of Unobserved States (PICRUSt) was utilized to predict the composition of functional genes within sample microbiomes to analyze differences between different species. A significant difference between species was defined as *p* < 0.05, whereas an extremely significant difference was defined as *p* < 0.01.

## 3. Results

### 3.1. Analysis of 16S rRNA Sequencing Data

A total of 2,285,650 effective reads were obtained from 10 fecal samples each from FSD (777,451 reads), FRD (777,411 reads), and FWLD (730,758 reads). In total, 1294 OTUs were obtained, including 1291, 1290, and 1283 in FSD, FRD, and FWLD, respectively. The three species shared 1278 OTUs, which accounted for 99.00% of the OTUs identified in FSD, 99.07% in FRD, and 99.61% in FWLD.

**Figure 1:**
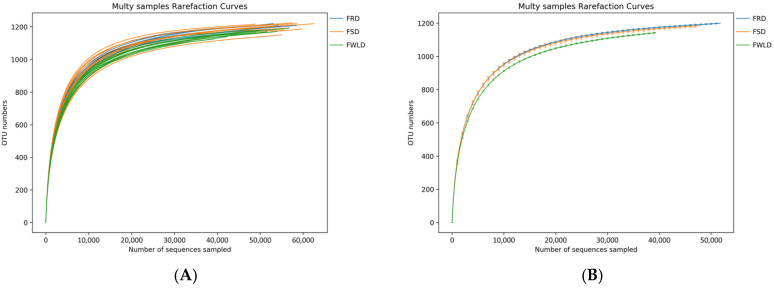
Rarefaction curves. The x-coordinate is the number of sequences sampled, and the y-coordinate is the number of observed OTUs. Each curve represents a sample (**A**) or a group (**B**), which is distinguished by different colors. The number of OTUs increases with the sequencing depth. When the curve gradually flattens, the number of detected OTUs does not increase with the expansion of extracted data, indicating that the amount of sequencing data is reasonable. (**A**) The rarefaction curves for each sample. (**B**) The rarefaction curves for each group.

**Figure 2:**
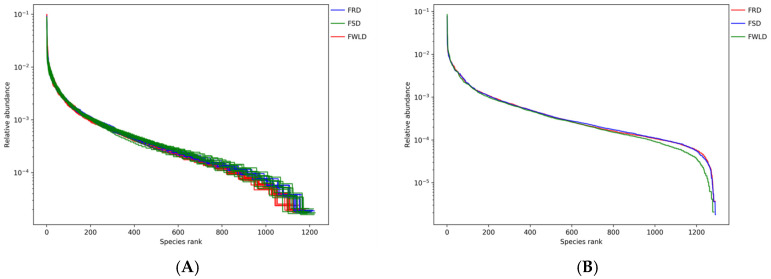
Rank–abundance curves. The rank–abundance curve is mainly used to explain the richness and evenness of species in the sample at the same time. The x-coordinate is the sequence number sorted by OTUs abundance from high to low, and the y-coordinate is the relative abundance of the corresponding OTUs. The larger the curve span along the x-coordinate, the richer the species composition of the sample. The flatter the curve on the y-coordinate, the higher the evenness of species composition of the sample. Different colors indicate different groups. (**A**) The rank–abundance curves for each sample. (**B**) The rank–abundance curves for each group.

The rarefaction curves (Figure 1) gradually flattened as the sequencing depth increased, indicating that the depth of the sampling protocol was sufficient to reasonably accurately reflect the microbial community of each sample. Figure 2 illustrates the rank–abundance curves of the samples. The larger the curve span along the x-coordinate, the more abundant the microorganisms in the samples. The smoother the curves on the y-coordinate, the higher the uniformity of the samples. According to the rank–abundance curves, the abundance and evenness of the intestinal microbiota in FWLD were lower than those in FSD and FRD, and there was little difference between those in FSD and FRD.

### 3.2. Analysis of Species Diversity

**Figure 3:**
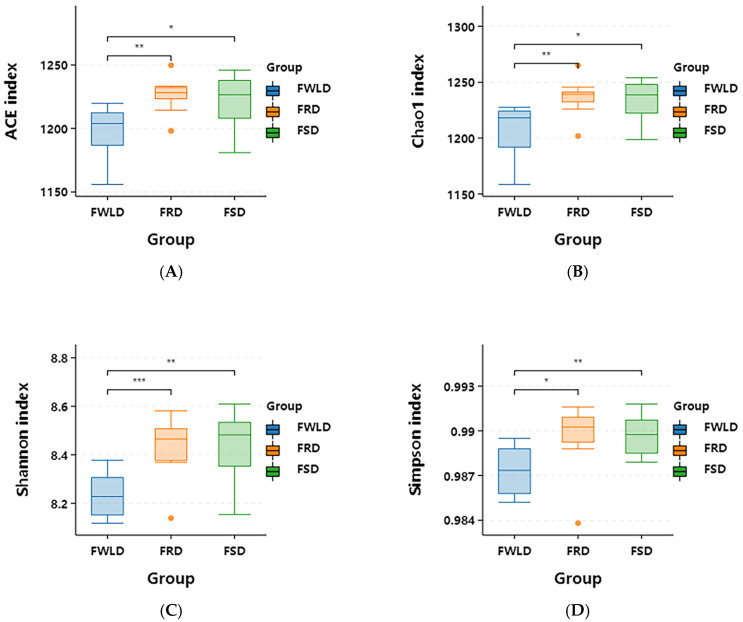
Box plot of alpha diversity indices. Inter-group statistical differences are analyzed using Student’s *t*-test. (**A**) ACE: an index that estimates the number of species in a community based on species abundance coverage. (**B**) Chao1: an index that estimates the total number of species in a sample using the Chao1 algorithm. (**C**) Shannon: an index that evaluates the species diversity of a community by integrating both species richness and evenness. (**D**) Simpson: an index used to quantitatively describe the biodiversity of a specific area. * *p* < 0.05, ** *p* < 0.01, *** *p* < 0.005.

As illustrated in Figure 3, the intestinal microbiota alpha diversity indices (ACE, Chao1, Shannon, and Simpson) indicated the following: (1) There were no significant differences in each alpha index between the intestinal microbiota of FSD and FRD (*p* > 0.05), indicating that their relative abundances and diversities in FSD and FRD were consistent with one another. (2) All alpha diversity indices of FRD were higher than those of FWLD. Furthermore, there were highly significant differences in the ACE, Chao1, and Shannon indices (*p* < 0.01) and significant differences in the Simpson index (*p* < 0.05). This indicates that the relative abundance and diversity of the intestinal microbiota in FRD were significantly higher than those in FWLD. (3) All alpha diversity indices of FSD were higher than those of FWLD. Furthermore, there were highly significant differences in the Shannon and Simpson indices (*p* < 0.01) and significant differences in the ACE and Chao1 indices (*p* < 0.05). This indicates that the relative abundance and diversity of the intestinal microbiota in FSD were significantly higher than those in FWLD.

**Figure 4:**
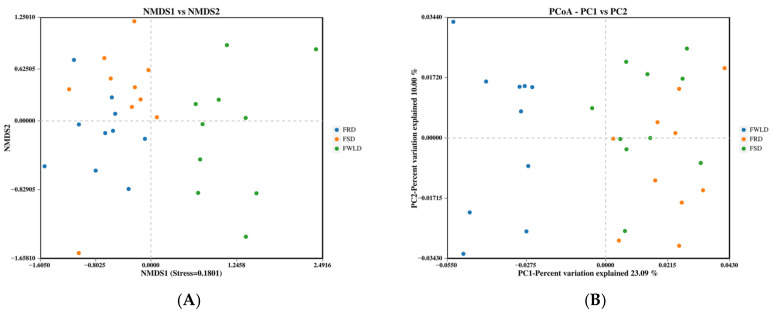
Beta diversity analyses based on an unweighted UniFrac distance metric. Each point represents a sample. The closer the distance between points, the more similar the community structure. Samples in the same group are represented by the same color. (**A**) Non-metric Multidimensional Scaling (NMDS) analysis plot. The distance between points represents the level of difference. A stress value < 0.2 indicates that the NMDS analysis is reliable. (**B**) Principal coordinate analysis (PCoA) plot. The axes represent the first and second principal coordinates (PC1, PC2). The percentages in axes indicate the proportion of total variance explained by each principal coordinate.

**Figure 5:**
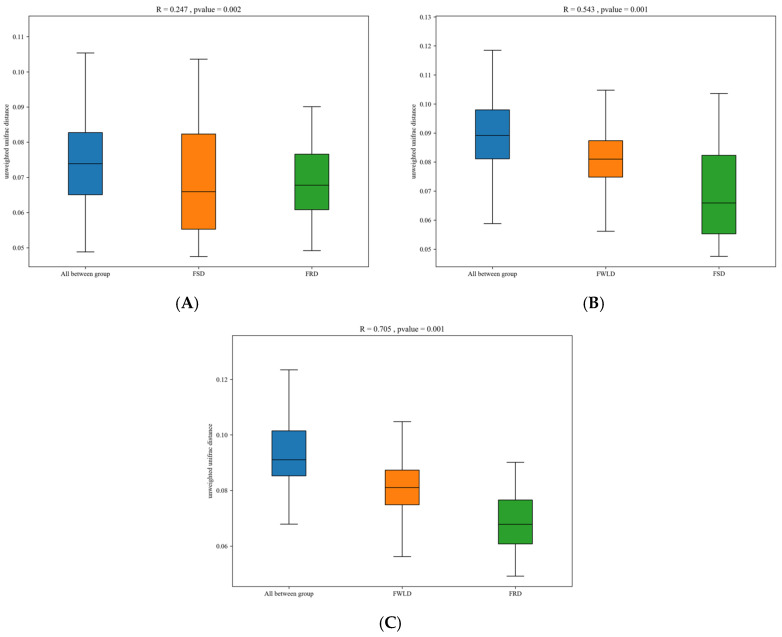
Analysis of Similarities (ANOSIM) analysis. The *x*-axis represents the grouping and the *y*-axis represents the distance calculated by unweighted UniFrac metric. The data in the box is the inter-group and intra-group distance, respectively. The range of R value is (−1, 1). R > 0 indicates that the difference between groups is greater than that within groups, and R < 0 indicates that the difference between groups is smaller than that within groups. The P value represents confidence level of the statistical analysis. *p* < 0.05 indicates a statistically significant difference. (**A**) Beta distance of FSD and FRD. (**B**) Beta distance of FWLD and FSD. (**C**) Beta distance of FWLD and FRD.

Beta diversity was calculated based on unweighted UniFrac, and NMDS and PCoA were utilized to analyze the community structure of the intestinal microbiota in the three host species (Figure 4). The points in Figure 4 represent individual samples; the closer the distance between points, the more similar their community structure. The results of the NMDS analysis were considered reliable if the stress value was less than 0.2, and the stress value for this analysis was 0.1801. Figure 4 illustrates that the samples from FSD and FRD were clustered together and clearly separated from the samples from FWLD. This indicates that the intestinal microbiota was less similar between FWLD and the other two groups and more similar between FSD and FRD. Furthermore, the results of ANOSIM (Figure 5) indicated that there were significant differences in intestinal microbiota composition between every pairwise comparison of the three deer, and the differences between species were greater than those within species (R > 0, *p* < 0.01).

### 3.3. Comparison of Bacterial Community Composition

The 1294 OTUs detected across all samples were classified into 14 phyla, 21 classes, 33 orders, 64 families, and 155 genera.

**Figure 6:**
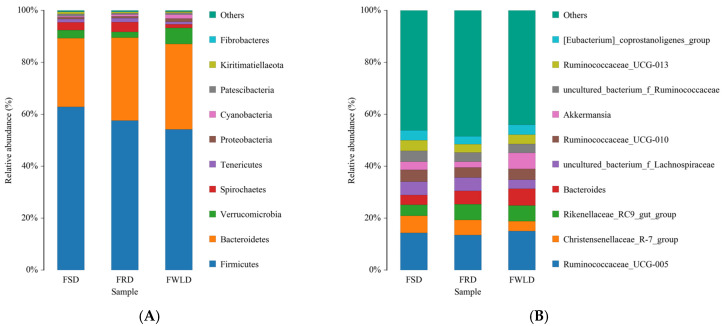
Intestinal microbiota composition. The x-coordinate represents the group name, and the y-coordinate represents relative abundance percentage. Different colors represent different species. The length of the color block represents the relative abundance ratio of the species. Only the top ten most abundant species are shown in the figure, while the rest are grouped into the “Others” category. (**A**) The top ten most abundant phyla in the intestinal microbiota among FSD, FRD, and FWLD groups. (**B**). The top ten most abundant genera in the intestinal microbiota among FSD, FRD, and FWLD groups.

The dominant phylum across all samples (Figure 6A, Table 2) was Firmicutes (FSD, 62.87%; FRD, 57.57%; and FWLD, 54.19%), followed by Bacteroidetes (FSD, 26.47%; FRD, 31.94%; and FWLD, 32.89%). Among the other phyla with high relative abundances, Verrucomicrobia had the highest relative abundance in FWLD (6.17%) and the lowest in FRD (2.27%). Cyanobacteria and Proteobacteria had the highest relative abundances in FWLD (1.54% and 1.25%, respectively) and the lowest in FSD (0.42% and 0.70%, respectively). Spirochaetes and Tenericutes had the highest relative abundance in FRD (3.70% and 1.52%, respectively) and the lowest in FWLD (1.44% and 0.95%, respectively).

The dominant genus (Figure 6B, Table 2) across all samples was *Ruminococcaceae_UCG-005* (FSD, 14.29%; FRD, 13.46%; and FWLD, 14.98%). Among the other genera with high relative abundances, *Christensenellaceae_R-7_group* and *uncultured_bacterium_f_ Ruminococcaceae* had the highest relative abundance in FSD (6.61% and 4.15%, respectively) and the lowest in FWLD (3.85% and 3.45%, respectively). *Ruminococcaceae_UCG-010*, *Ruminococcaceae_UCG-013*, and *[Eubacterium]_coprostanoligenes_group* had the highest relative abundances in FSD (4.60%, 4.15%, and 3.82%, respectively) and the lowest in FRD (3.89%, 3.13%, and 3.07%, respectively). *Uncultured_bacterium_f_Lachnospiraceae* had the highest relative abundance in FRD (5.16%) and the lowest in FWLD (3.44%). *Bacteroides* and *Rikenellaceae_RC9_gut_group* had the highest relative abundance in FWLD (6.47% and 6.03%, respectively) and the lowest in FSD (3.69% and 4.27%, respectively). *Akkermansia* had the highest relative abundance in FWLD (6.17%) and the lowest in FRD (2.27%).

**Figure 7:**
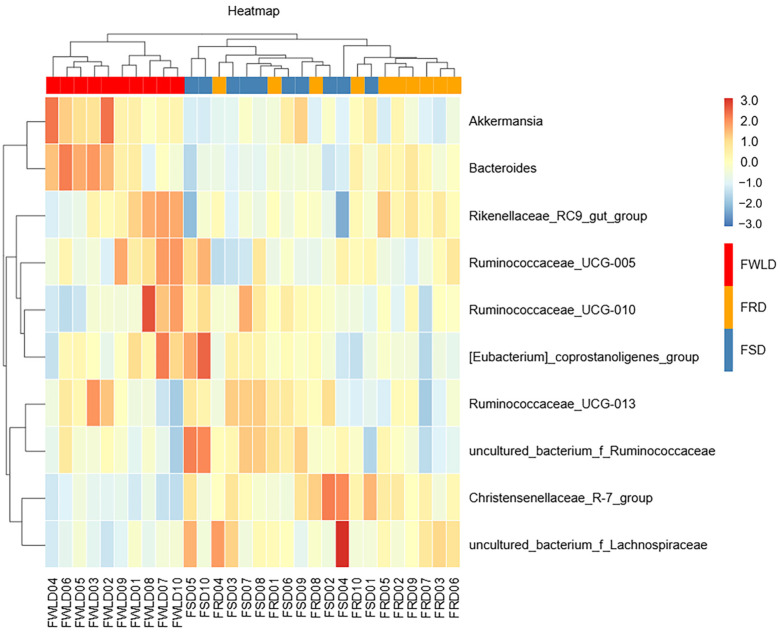
Heatmap of the 10 most abundant genera. Each row represents a genus and columns represent the 30 individual samples. The left side of the graph is the genus clustering tree and top is the sample clustering tree. The corresponding values of the heatmap are the *Z* values obtained by normalizing the relative abundance of genera on each row. The color gradient from blue to red indicates a low to high relative abundance. The vertical clustering indicates the similarity in the richness of different species among samples. The closer the distance between two species, the shorter the branch length, indicating greater similarity in richness between the two species. Horizontal clustering indicates the similarity of species richness in different samples. The closer the distance between two samples, the shorter the branch length, indicating greater similarity of species richness between the two samples.

A heatmap of the relative abundances of OTUs at the genus level is shown in Figure 7. After log normalization of the OTUs, the 10 most abundant genera were selected for clustering. Drawing was then performed using an R heatmap. Each color block in the heatmap represents the relative abundance of one genus in one sample, with samples arranged horizontally and OTUs arranged vertically. Heatmap clustering indicated that the intestinal microbiota in FWLD clustered into a separate region compared to those of FSD and FRD, and there was no significant separation between FSD and FRD.

### 3.4. Comparison of Differences in Intestinal Microbiota in Different Groups

**Figure 8:**
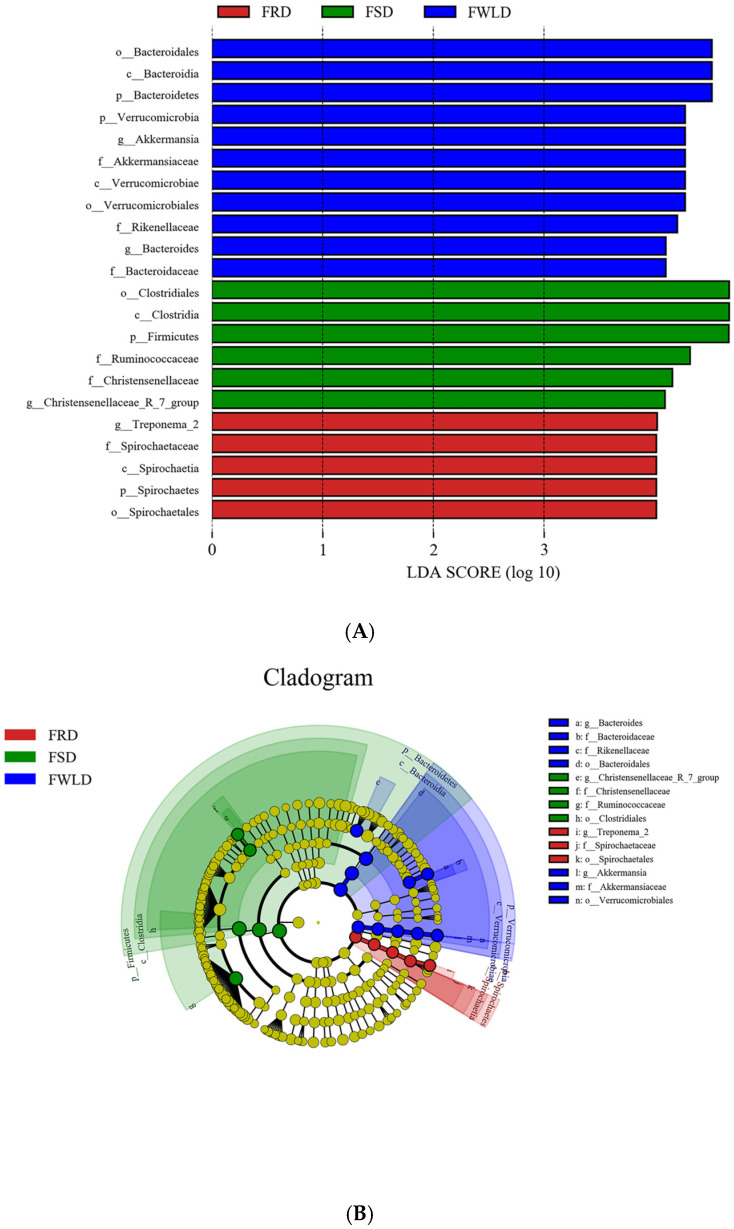
LEfSe analysis. (**A**) Species with significant difference that have an LDA score greater than the estimated value; the default score is 4.0. The length of the histogram represents the LDA score. (**B**) The cladogram diagram shows the microbial species with significant differences in the three groups, and the species classification at the level of phylum, class, order, family, and genus shown from the inside to the outside. The red, green, and blue nodes in the phylogenetic tree represent microbial species that play an important role in the FRD, FSD, and FWLD groups, respectively. Yellow nodes represent species with no significant differences.

Via LEfSe analysis (LDA > 4.0), significant differences in the microbial communities were identified among the three host species. As shown in Figure 8, there were 22 significant differences identified in the intestinal microbiota among hosts. In FSD, the relative abundances of Firmicutes, Clostridia, Clostridiales, Ruminococcaceae, Christensenellaceae, and *Christensenellaceae_R-7_group* were higher than those in FRD or FWLD. The relative abundances of Spirochaetes, Spirochaetia, Spirochaetales, Spirochaetaceae, and *Treponema_2* were higher in FRD than in FSD or FWLD. The relative abundances of Verrucomicrobia, Verrucomicrobiae, Verrucomicrobiales, Akkermansiaceae, *Akkermansia*, Bacteroidetes, Bacteroidia, Bacteroidales, Bacteroidaceae, *Bacteroides*, and Rikenellaceae were higher in FWLD than in FSD or FRD.

### 3.5. Analysis of PICRUSt Function Prediction

**Figure 9:**
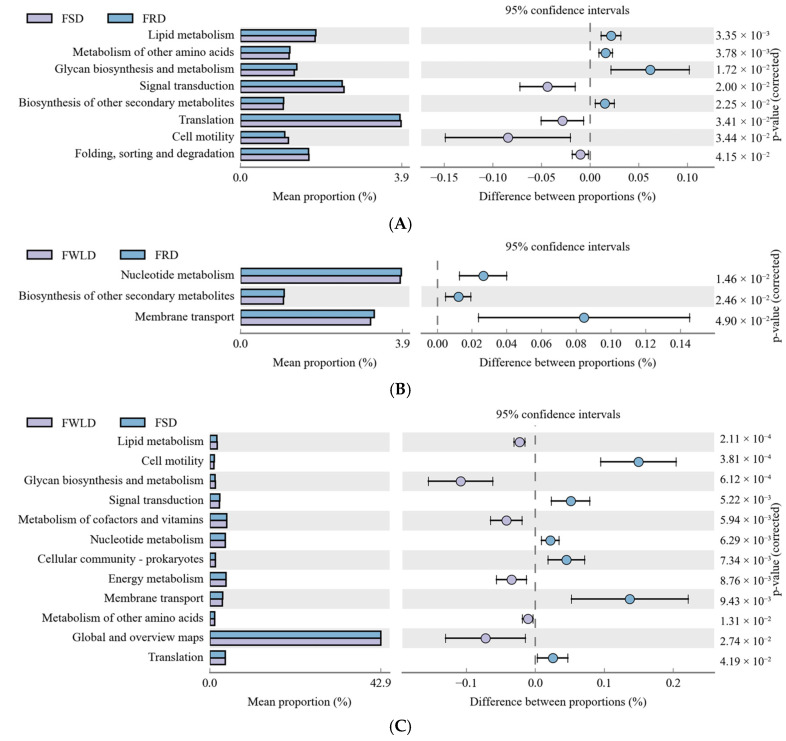
Phylogenetic Investigation of Communities by Reconstruction of Unobserved States (PICRUSt) analysis. Variance analysis of the KEGG metabolic pathways in the second level. The graphs show the abundance ratio of different functions in two groups of samples. The middle shows the difference between proportions of functional abundance in the 95% confidence interval, and the value at the rightmost is the P value. *p* < 0.05 represents the significant difference. (**A**) The abundance ratio of different functions between FSD and FRD. (**B**) The abundance ratio of different functions between FWLD and FRD. (**C**) The abundance ratio of different functions between FWLD and FSD.

Differences in the functional gene composition and function of each bacterial community was inferred using PICRUSt. Using the Kyoto Encyclopedia of Genes and Genomes (KEGG) database, differences in metabolic pathways in the microbial communities of each host species at the second level were compared (Figure 9; only functional relative abundances greater than 1% were evaluated).

In the functional categories of “Lipid metabolism”, “Glycan biosynthesis and metabolism”, and “Metabolism of other amino acids”, the relative abundances of the intestinal microbiota in FRD and FWLD were significantly higher than that in FSD, but there was no significant difference between the first two groups. In the functional category of “Biosynthesis of other secondary metabolism”, the relative abundance of the intestinal microbiota in FRD was significantly higher than those in FSD and FWLD, and there was no significant difference between the latter two groups. In the functional category of “Nucleotide metabolism”, the relative abundances of the intestinal microbiota in FRD and FSD were significantly higher than that in FWLD, but there was no significant difference between the first two groups. In the functional categories of “Metabolism of cofactors and vitamins” and “Energy metabolism”, the relative abundance of the intestinal microbiota in FWLD was significantly higher than that in FSD, and no significant difference was found with that in FRD.

## 4. Discussion

Sometimes referred to as “the second genome” of its host, the intestinal microbiota exhibits a variety of functions and plays an important role in the digestive physiology, metabolism, immune system regulation, resistance to pathogens, and many other physiological processes of its host. The complex digestive system of ruminants leads to distinct microbial communities in both the rumen and feces. For the three deer species (sika deer, red deer, and white-lipped deer), rumen contents sampling presents significant practical challenges. Previous studies have demonstrated that fecal microbiota can represent intestinal microbiota composition to a certain extent. Therefore, fresh feces samples were selected as the experimental material in the present study.

Alpha diversity indices indicated that the relative abundance (ACE, Chao1) and diversity (Shannon, Simpson) of the intestinal microbiota in FSD and FRD were significantly higher than those in FWLD, while there was no significant difference between the former two host species. Beta diversity analysis revealed significant differences in the intestinal microbiota community structure of the three deer species, and the community structure of FWLD was less similar to that of the other two species. The intestinal microbiota in FWLD differed significantly from those of FRD and FSD. Some studies have demonstrated that closely related species have more similar intestinal microbiota [38,39,40,41]. According to taxonomic studies, white-lipped deer and sika deer diverged from their common ancestor, sambar deer (*Rusa unicolor*), while red deer diverged from sika deer, indicating that sika deer and red deer are more closely related [42,43,44]. Meanwhile, female white-lipped deer (body length 110–120 cm, shoulder height 120–130 cm, and body weight approximately 150 kg) are similar in build to female red deer (body length 120–140 cm, shoulder height 110–130 cm, and body weight approximately 200 kg), albeit slightly smaller. However, their body size is almost twice that of female sika deer (body length 75–90 cm, shoulder height 80–95 cm, body weight approximately 80 kg). It may be that the intestinal microbiota of closely related sika deer and red deer was similar due to their shorter phylogenetic distance from one another, whereas the more distantly related white-lipped deer exhibited greater differences in their intestinal microbiota composition compared to the two former species. Therefore, the phylogenetic relationships between the three deer species appear to exhibit a more significant impact on their intestinal microbiota than body size.

At the phylum level, Firmicutes and Bacteroidetes dominated the intestinal microbiota of all three deer species. This is consistent with findings for other ruminant animals, such as blue sheep (*Pseudois nayaur*) [11], forest musk deer (*Moschus berezovskii*) [13,15], and Père David’s deer (*Elaphurus davidianus*) [17]. Firmicutes are primarily known for degrading cellulose and synthesizing volatile fatty acids (VFAs) [45]. Bacteroidetes are primarily known for degrading carbohydrates, proteins, and other substances, thus promoting the development of the host intestinal immune system [46]. The ratio of Firmicutes to Bacteroidetes (F/B ratio) may reflect the ability of the host to obtain energy from food. The higher the ratio, the higher the efficiency of energy acquisition [18]. In this study, the diet of the deer primarily consisted of corn straw silage, which has a high fiber and low protein content. The F/B ratios of FSD, FRD, and FWLD were 2.38, 1.80, and 1.65, respectively, reflecting the adaptability of the three deer species to this feed.

At the genus level, *Ruminococcceae_UCG-005* was most abundant in the intestinal microbiota of all three deer species. It is the main bacterial genus capable of degrading cellulose and its abundance has been observed to increase with increasing dietary fiber consumption [47]. *Christensenellaceae_R-7_group*, *Rikenellaceae_RC9_gut_group*, and *Bacteroides* were the second most abundant bacterial genera in FSD, FRD, and FWLD, respectively. *Christensenellaceae_R-7_group* is capable of decomposing and fermenting sugars and proteins in foods which can then be utilized by the host [48]. *Rikenellaceae_RC9_gut_group* is capable of promoting host lipid metabolism [49]. *Bacteroides* are involved in various metabolic activities, such as carbohydrate fermentation, bile acid and steroid biotransformation, and the utilization of nitrogenous substances in the host colon. In addition, acetate, propionate, and other products produced by *Bacteroides* metabolism are effective mediators of the host inflammatory response [50]. These genera are beneficial intestinal bacteria. No pathogenic bacteria were found in this study, indicating that the intestinal microecosystems of the three deer species reared on this farm were healthy. Notably, *Christensenellaceae_R-7_group* was significantly more abundant in the intestinal microbiota of FSD than in those of FRD and FWLD. The relative abundance of *Treponema_2* in FRD was significantly higher than those in FSD and FWLD. *Treponema_2* belongs to the family Spirochaetaceae and is a common potential pathogen [51]. An increase in its relative abundance may lead to diarrhea, acute myocardial ischemia, digital dermatitis, and other symptoms in the host [52,53,54]. However, *Treponema bryantii* sp. nov. [55] and *T. succinifaciens* sp. nov. [56] have been observed to be beneficial to their host. The relative abundance of *Akkermansia* in FWLD was significantly higher than in FSD and FRD. *Akkermansia* belongs to the genus Verrucomicrobia and is a mucin-degrading bacterium found in the gut. It mainly utilizes mucin as a carbon and nitrogen source, fermenting it to produce acetate, propionate, and other substances. These products have been suggested to be able to regulate the immune response, lipid metabolism, and other biological functions of the host while inhibiting host inflammatory responses to maintain host intestinal health [57].

The results of the PICRUSt function prediction indicated that the abundance of functional genes related to nutrient metabolism pathways in the FSD intestinal microbiota was significantly lower than those found in FRD and FWLD, whereas the abundance of functional genes related to intestinal absorption in FWLD was significantly lower than those in FSD and FRD. The intestinal microbiota is closely associated with host digestion and absorption. It can assist the host in decomposing carbohydrates, proteins, and cellulose into an absorbable form [58]. The results of this study indicated that, under the same feeding environment, the efficiency of food absorption and utilization of FWLD may be lower than that of FSD and FRD. This may be because the white-lipped deer living in the Qinghai–Tibet Plateau are not as adaptable to the feeding mode and food composition (mainly corn straw) utilized in this study as the other two deer species. In addition, the intestinal microbiota in FSD exhibited lower metabolic functionality than those in FRD and FWLD. This may be because sika deer are small- to medium-sized Cervidae, whereas red deer and white-lipped deer are considered large Cervidae. The smaller size of the sika deer leads to a decrease in the metabolic function of their intestinal microbiota.

To date, there have been many studies on the intestinal microbiota of herbivores. Hu et al. studied the gut microbial communities in forest musk deer and alpine musk deer (*M. chrysogaster*). Although the living environment of the two musk deer was similar, there were differences in the types of feed leaves [13]. Different dietary compositions could affect the composition of intestinal microbiota, so these differences might not be due to differences in species. Sun et al. compared the differences in intestinal microbiota between European mouflon sheep (*Ovis orientalis musimon*) and blue sheep at different altitudes [11]. They focused on discussing the impact of altitude on the host intestinal microbiota, but ignored the similarities and differences between the intestinal microbiota of two sheep at the same altitude. White-tailed deer (*Odocoileus virginianus*) is a common breeding deer species in North America. Some studies explored the links between the intestinal microbiota of white-tailed deer and infectious diseases. The results indicated that the differences of intestinal microbiota compositions may be linked to pathogenesis [16,59]. Zhang et al. examined the differences in intestinal microbiota between the Père David’s deer populations in the Beijing and Shishou, Hubei Province. The results indicated that during the ex situ conservation process of Père David’s deer, their food sources may change, resulting in differences in the intestinal microbiota. Although noticing food sources were related to the intestinal microbiota composition and diversity, they ignored the inconsistencies of living environments in Beijing and Shishou [17]. In this study, the living environment and food were strictly the same between the three deer species. The only possible factor that could affect the composition of the intestinal microbiota of the three deer was the difference in species; this was the uniqueness of this study.

## 5. Conclusions

In summary, in this study, 16S rRNA high-throughput technology was employed to conduct research on the intestinal microbiota of three deer species. Under the identical feeding environment and feed supply conditions, the phylogenetic relatedness of the three host deer significantly influenced their intestinal microecosystems. Specifically, the alpha diversities of the intestinal microbiota of sika deer and red deer were significantly higher than those of white-lipped deer. The higher the diversity of the intestinal microbiota, the more complex the composition, the stronger the ability to resist external disturbances, and the greater the adaptability. Sika deer and red deer demonstrated stronger adaptability. The intestinal microbial structure of sika deer and red deer was similar, and it differed significantly from that of white-lipped deer, suggesting that sika deer and red deer have similar feeding habits. Therefore, considering the impact of the phylogenetic relationship of the three deer species on the intestinal microbiota, it is necessary to enhance the nutritional level of the feed for white-lipped deer and augment its ability to acquire energy. In the future, more advanced analytical techniques, sampling methods, and sample types will be applied in the research on the intestinal microbiota of deer. Simultaneously, the effects of different diets and environmental factors on the intestinal flora of these deer species will be explored. These studies will provide a scientific theoretical basis for the development of deer breeding in China.

## Figures and Tables

**Table 1:** Composition and nutritional level in concentrated feed of diet for three deer species.

Items	Content (%)
Composition	
Corn meal	48.9
Soybean meal	23.0
Heat-treated soybean	14.0
Wheat bran	10.0
NaCl	1.5
Mineral premix	2.6
Total	100.0
Nutritional level	
GE (MJ/kg)	16.89
ME (MJ/kg)	12.25
CP	20.00
Ca	0.99
P	1.02

GE, gross energy; ME, metabolizable energy; CP, crude protein.

**Table 2:** Relative abundance of the top ten most abundant phyla and genera in the intestinal microbiota of three host species.

Taxon	Name	Relative Abundance (%)		
		FSD	FRD	FWLD
Phylum	Firmicutes	62.87	57.57	54.19
	Bacteroidetes	26.47	31.94	32.89
	Verrucomicrobia	3.13	2.27	6.17
	Cyanobacteria	0.42	0.66	1.54
	Proteobacteria	0.70	0.74	1.25
	Spirochaetes	2.94	3.70	1.44
	Tenericutes	1.26	1.52	0.95
	Patescibacteria	0.94	0.58	0.67
	Kiritimatiellaeota	0.76	0.50	0.41
	Fibrobacteres	0.14	0.22	0.11
Genus	*Ruminococcaceae_UCG-005*	14.29	13.46	14.98
	*Christensenellaceae_R-7_group*	6.61	5.87	3.85
	*Rikenellaceae_RC9_gut_group*	4.27	6.01	6.03
	*Bacteroides*	3.69	5.13	6.47
	*uncultured_bacterium_f_Lachnospiraceae*	5.15	5.16	3.44
	*Ruminococcaceae_UCG-010*	4.60	3.89	4.20
	*Akkermansia*	3.13	2.27	6.17
	*uncultured_bacterium_f_ Ruminococcaceae*	4.15	3.54	3.45
	*Ruminococcaceae_UCG-013*	4.15	3.13	3.60
	*[Eubacterium]_coprostanoligenes_group*	3.82	3.07	3.79

Taxon, the taxonomic ranks of the microbial species; Name, the names of the microbial species; Relative Abundance, the relative abundance percentage of the microbial species; FSD, female sika deer; FRD, female red deer; FWLD, female white-lipped deer.

## Data Availability

The sequencing data generated in our study have been deposited in the SRA database (BioProject ID: PRJNA1059233).
